# Potential impact of urolithin A on pathways relevant to sleep health: a mini review

**DOI:** 10.3389/fnut.2026.1779855

**Published:** 2026-03-04

**Authors:** Ana Clara da C. Pinaffi-Langley, Faris M. Zuraikat, Marie-Pierre St-Onge, Andriy Yabluchanskiy

**Affiliations:** 1 Department of Neurosurgery, College of Medicine, University of Oklahoma Health Campus, Oklahoma City, OK, United States; 2 Division of General Medicine and Center of Excellence for Sleep & Circadian Research, Department of Medicine, Columbia University Irving Medical Center, New York, NY, United States; 3 Institute of Human Nutrition, Vagelos College of Physicians & Surgeons, Columbia University Irving Medical Center, New York, NY, United States

**Keywords:** gut microbiota, polyphenol-derived microbial metabolites, polyphenols, sleep, urolithin A

## Abstract

Sleep is vital to human health, and poor sleep health has been associated with numerous chronic conditions, including cardiovascular disease, obesity, depression, and type 2 diabetes. Recently, an emerging area of research has focused on the relationship between dietary polyphenols and sleep health. This connection may be mediated by the gut microbiota and polyphenol-derived microbial metabolites, which also exert biologically relevant effects. One such metabolite, urolithin A, has been shown to improve mitochondrial function, muscle strength, and inflammation in humans. However, its potential effect on sleep remains unexplored. Thus, this mini review aimed to summarize the current evidence on the effect of urolithin A on sleep-relevant pathways and to explore the possible mechanisms underlying this effect. Although no study directly investigating the effect of urolithin A on sleep outcomes was identified, our search found four preclinical studies that included outcomes relevant to sleep. These studies provide mechanistic plausibility for the relationship between urolithin A and sleep health through direct and indirect mechanisms such as modulation of the central clock, protection against neuroinflammation caused by sleep deprivation, and modulation of the gut microbiota. However, to elucidate the direct effect of urolithin A on sleep, studies with specific sleep measures such as electroencephalogram, actigraphy, and/or polysomnography are still required. Taken together, this represents a novel direction in polyphenol-derived microbial metabolite research with many opportunities for future research.

## 1 Introduction

Healthful sleep-encompassing its multiple dimensions of regularity, satisfaction, duration, continuity, timing, architecture, (absence of) disorders, and daytime functioning-is vital to human health (1, 2). Several meta-analyses have reported associations between poor sleep health, especially short sleep duration, and an increased risk of type 2 diabetes mellitus (3, 4), cardiovascular disease (5, 6), cognitive impairment and dementia (7–9), and all-cause mortality (10). Despite this strong evidence, poor sleep health is widespread across age ranges and populations. Data from nationally representative surveys show that almost 1 in 3 US adults report sleep disturbances or sleep deprivation (11), while almost 1 in 2 report poor sleep satisfaction (12). These statistics underscore the need to develop novel, actionable strategies to help individuals achieve regular, restorative sleep.

Many factors influence sleep health, including social (e.g., socioeconomic status and work obligations), and individual factors (e.g., lifestyle choices). Lifestyle determinants of sleep are more readily amenable to modification. For example, dietary habits-such as adhering to a healthful diet rich in vegetables, fruits, legumes, unsaturated fats, and whole grains-are strongly correlated with sleep health (13–15). Healthful dietary patterns shown to predict sleep outcomes tend to be rich in polyphenols, secondary plant metabolites with biological effects relevant to human health (16). Thus, an emerging research avenue garnering interest is the effect of polyphenols on sleep health (17–19). While studies have focused on associations of polyphenol intake with sleep outcomes, it is notable that this connection may be mediated by the gut microbiota. As polyphenols are poorly bioavailable, they accumulate in the colon and are extensively metabolized by the resident gut bacteria, generating polyphenol-derived microbial metabolites. These metabolites exert biologically relevant effects of their own and have greater bioavailability than their parent polyphenolic compounds (20), representing a likely mediator of polyphenol’s effect on health outcomes, including on sleep health. One such metabolite, urolithin A, has been shown to improve mitochondrial function and inflammation in humans (21–24). The potential effect of urolithin A on sleep, however, is largely unexplored. Therefore, this mini review aims to summarize the current evidence surrounding the effect of urolithin A on biological pathways relevant to sleep health and explore possible mechanisms by which this polyphenolic metabolite could impact sleep, thereby highlighting future opportunities for research.

## 2 Urolithin A: brief overview

Urolithin A is an organic compound belonging to the benzo-coumarin class and a member of the broader family of urolithins. Urolithins are microbial metabolites derived from ellagitannins and ellagic acid, polyphenols naturally present in foods such as berries, pomegranate, and nuts (25). These parent polyphenols exhibit low bioavailability and accumulate in the colon, where they are catabolized by specific gut bacterial species to generate urolithins, including urolithin A. Although the identities of the bacteria involved in urolithin biosynthesis are only partially known, members of the *Gordonibacter* and *Ellagibacter* genera have been implicated in this process (25, 26).

The biosynthesis of urolithin A comprises several catabolic reactions happening mainly in the colon. The first step involves ester hydrolysis to release ellagic acid moieties from their associated sugars. Once released, ellagic acid undergoes cleavage of a carbon–oxygen bond, resulting in lactone ring opening, followed by a decarboxylation reaction that yields the first urolithin intermediate. Subsequent de-hydroxylation reactions generate urolithins with varying degrees of hydroxylation, with urolithin A being a dihydroxy urolithin (20, 27).

Following their production in the colon, urolithins are absorbed and transported to the liver, where they undergo phase II biotransformation prior to entering the systemic circulation. One of these phase II metabolites, urolithin A-glucuronide, represents urolithin A’s predominant circulating metabolite. In healthy adults, plasma concentrations of urolithin A-glucuronide reach a peak of approximately 110 ng/mL and 30 ng/mL at 24 h post consumption of pomegranate juice (107 mg of ellagitannins and ellagic acid) (28) and frozen red raspberry beverage (29 mg of ellagitannins and ellagic acid) (29), respectively. The pharmacokinetics of urolithin A also vary considerably depending on whether the intake was food-based (i.e., foods rich in ellagitannins) or supplement-based (i.e., isolated urolithin A). Plasma urolithin A and its phase-II conjugates reach peak concentration about 6 h post-intake when supplemented directly, whereas peak concentration happens about 24 h post-intake when given via food (21, 25, 28).

Individuals are commonly classified as urolithin A producers (metabotype A), urolithin A, B, and isourolithin A producers (metabotype B), or non-producers (metabotype 0), according to their urolithin production following an acute dose of ellagitannins and/or ellagic acid (25). However, this classification is not static and can change over time due to factors such as age and dietary habits. In fact, studies using foods rich in ellagitannins have reported metabotype conversion from baseline to endpoint (30). Interindividual variability is high even when circumventing the gut microbiota with isolated metabolites, with plasma urolithin A-glucuronide levels in healthy adults exhibiting a 49% relative coefficient of variation after a standardized dose of 500 mg of urolithin A (28). This is likely due to interindividual differences in absorption, entero-hepatobiliary circulation, and other factors associated with bioavailability. Among adults aged 20–72 years, the metabotype distribution has been reported to be approximately 50–55% metabotype A, 35–40% metabotype B, and 7–10% metabotype 0 (31, 32). Importantly, these studies were conducted in the Mediterranean region (Spain) and, given that genetic and environmental factors both influence metabotype, it is unknown whether these reported metabotype distributions are generalizable to other populations.

## 3 Urolithin A and sleep: current evidence

For this mini-review, we utilized a hypothesis-based scoping review method where we conducted a search on PubMed, Scopus and Ovid utilizing the terms “urolithin A” and “sleep,” “urolithin A” and “circadian,” and “urolithin A” and “serotonin or tryptophan.” Our inclusion criteria were (1) original research articles, (2) published in English, (3) utilizing isolated urolithin A (no mixtures or parent polyphenols) and (4) including at least one outcome relevant to sleep or sleep-related biological mechanisms, with no constraints on publication date. Our initial search produced 81 publications. After removing duplicates and screening publications according to inclusion criteria (for a flow diagram, see Supplementary Figure 1), five articles remained (Table 1). All were pre-clinical studies performed in mice or rats.

**Table 1:** Summary of studies on the effect of urolithin A on pathways relevant to sleep health.

First author (year)	Organism	Intervention	Dose	Control	Duration	Findings
Du (2024) (33)	Female C57BL/6J mice	Pre-treatment with UA prior to induction of inflammation	20 mg/kg/day in oral gavage	0.5% CMC and 0.1% TW80 daily	1 week	Upregulation of clock-related genes (*Bmal1*, *Per2*, *Clock*, *Cry1*, *Per3*) in the SCN
Zhu (2024) (37)	2-month-old male C57BL/6J mice	Pre-treatment with UA prior to 48-h SD; treatment continued during SD	50 mg/kg/bw in oral gavage	Saline plus no SD, saline plus SD, and caffeine plus SD (10 mg/kg/bw)	1 week	Pre-treatment with UA suppressed inflammation, microbial dysbiosis, and fatigue caused by SD
Zhou (2024) (35)	6-month-old Sprague Dawley rats	Treatment with UA during a TSI condition to simulate microgravity	20 mg/kg/day in oral gavage	PBS alone	28 days	Treatment with UA mitigated dysfunctions in core body temperature, heart rate, and locomotor-activity rhythms caused by TSI
Misrani (2023) (38)	3-month-old and 12-month-old C57BL/6J mice	Pre-treatment with UA prior to 48-h SD	2.5 mg/kg or 10 mg/kg given i.p.	Saline plus no SD, saline plus SD, UA plus no SD	1 week	Pre-treatment with UA suppressed neuroinflammation, mitochondrial dysfunction, and cognitive deficits caused by SD
Haraguchi (2022) (34)	MEFs and SCN explants from PER2::LUC mice	Treatment with UA after dexamethasone stimulation	10–100 μM in MEFs, 5 or 50 μM in SCN explants	0.1% DMSO	Acute (30 min) or up to 6 days	Treatment with UA delayed the rhythmic expression of *Per2* in the SCN and prolonged the periodicity of *Per2* expression in MEFs in a dose-dependent manner

CMC, carboxymethyl cellulose; i.p., intraperitonially; MEF, mouse embryonic fibroblasts; PBS, phosphate buffer saline; SCN, suprachiasmatic nucleus; SD, sleep deprivation; TSI, tail suspension and isolation; UA, urolithin A.

Two studies (33, 34) reported effects of urolithin A on the central clock. Du et al. (33) analyzed the suprachiasmatic nucleus (SCN) of mice that underwent 1 week of urolithin A (20 mg/kg/day) or vehicle pre-treatment prior to dextran sulfate sodium-induced intestinal inflammation to study the effects of urolithin A on the expression of central clock genes. They reported a significant increase in the expression of *Clock*, *Cry1*, and *Bmal1* genes. However, the significance of this effect is unclear as the authors did not include comparator groups without dextran sulfate sodium treatment, which would be needed meaningfully assess the effect of the treatment on circadian rhythmicity. Additionally, there was no assessment of protein levels downstream of changes in gene expression. Another experiment from Haraguchi et al. (34) used a mouse model that allows the visualization of *Per2* expression rhythmicity via bioluminescence (PER2::LUC) to investigate the effect of varying doses of urolithin A on *Per2* expression in mouse embryonic fibroblasts and cultured SCN explants. Among tested dose ranges of 10–100 μM, treatment with lower doses of urolithin A (10–20 μM) increased *Per2* first and second peak amplitude, delayed first peak time, and prolonged period length in mouse embryonic fibroblasts. Similarly, in the SCN, a low dose of urolithin A treatment (5 vs. 50 μM) delayed the first peak time but had no effect on amplitude or period length. It is notable, however, that all urolithin A doses used in this study would exceed physiological thresholds for humans; peak plasma levels of urolithin A and its metabolites only reach about 110 ng/mL (equivalent to 1.2 μM) even after direct supplementation with 500 mg of urolithin A in healthy adults (28). Although these mouse models provide evidence that urolithin A can modulate the molecular machinery driving circadian rhythms, they have not evaluated the potential downstream favorable effects on sleep. Furthermore, additional experiments under forced desynchrony conditions are necessary to assess whether urolithin A can restore rhythmicity in adverse conditions.

Zhou et al. (35) investigated whether urolithin A can mitigate circadian rhythm disruption caused by simulated microgravity and isolation associated with spaceflight. They utilized a 30-degree tail suspension and isolation (TSI) model that kept the animals’ hindlimbs off the ground for 28 days to simulate microgravity. They reported that animals in the TSI group had lower circadian amplitude of core body temperature, heart rate, and locomotor-activity rhythms relative to control animals. They also reported that the SCN of rats in the TSI group had increased and decreased Rev-Erbα and Bmal1 protein levels, respectively, in addition to neuronal mitochondrial dysfunction, compared with that of rats in the control group. Rev-Erbα plays an important role in the regulation of the circadian oscillator, targeting *Bmal1* and *Clock* directly to downregulate their transcription (36). When treated with urolithin A during TSI conditions, the circadian amplitude of core body temperature, heart rate, and locomotor-activity were restored to control conditions, and Rev-Erbα and Bmal1 protein levels in the SCN were decreased and increased, respectively.

The other two available studies (37, 38) evaluated whether urolithin A can counter adverse effects of acute sleep deprivation. Zhu et al. (37) tested the protective effects of urolithin A on sleep deprivation-induced fatigue and gut dysbiosis in male mice, including negative (regular sleep schedule, vehicle treatment) and positive (caffeine treatment) controls. Compared with negative controls, animals that received urolithin A treatment had preserved exercise capacity, measured as grip strength, and time to fatigue on a rota-rod test. Interestingly, these effects of urolithin A were comparable to those of caffeine (positive control), a well-known ergogenic aid (39). Sleep-deprived animals that received urolithin A vs. vehicle-treated controls also had a better inflammatory and oxidative stress profile, with significantly higher levels of glutathione peroxidase and lower levels of malondialdehyde in the liver as well as significantly lower circulating levels of C-reactive protein, interleukin-6, and tumor necrosis factor-*α*. With respect to the gut microbiota, treatment with urolithin A increased the abundance of commensal bacteria (*Lactobacillus*, *Lachnospiraceae*) and suppressed the proliferation of pathogenic bacteria (*Clostridia*, *Candidatus*) compared with vehicle-treated, sleep-deprived controls.

In a separate series of experiments of urolithin A treatment in the context of imposed sleep disturbance, Misrani et al. (38) investigated whether urolithin A could attenuate declines in cognition and brain health of animals. The authors reported that treatment with urolithin A preserved performance on a spatial learning and memory test (Morris water maze) after sleep deprivation relative to vehicle-treated sleep-deprived controls. They also reported that treatment with urolithin A prior to sleep deprivation blunted the overactivation of glial cells, attenuated the increase in pro-inflammatory cytokines (IL-6, TNF-*α*), and maintained mitochondrial dynamics and morphology integrity in the hippocampus, a brain region highly susceptible to sleep deprivation-induced deficits (40). The authors included two different urolithin A doses (2.5 and 10 mg/kg of body weight) but reported no evidence of a dose–response effect. There was also no beneficial effect of urolithin A treatment in the absence of sleep deprivation, and its effects on young and old mice were comparable.

The urolithin A doses used in the aforementioned mouse studies (37, 38) correspond to human-equivalent doses of approximately 14–284 mg/day (for a 70-kg human, based on body surface area allometric scaling). These doses are realistic to humans as isolated urolithin A has been shown to be safe and well-tolerated in middle-aged and older adults in doses up to 2,000 mg/day (21). Although these studies did not investigate the effects of urolithin A on sleep directly, they have shown that urolithin A may help mitigate the adverse effects of sleep deprivation, with potential to improve daytime function and perceived well-being in individuals with poor sleep health. However, major limitations remain: (1) only preclinical studies conducted in cell culture or animal models were identified for this review, and (2) the identified studies were not explicitly designed to measured changes in sleep. Thus, preclinical and clinical studies designed to evaluate sleep health are critically needed to address these gaps and elucidate the role of urolithin A in promoting good sleep health.

## 4 Urolithin A and sleep: potential mechanisms

Currently, we have identified no studies directly investigating the effect of urolithin A on sleep measures such as duration, efficiency, continuity, or timing. However, we identified emerging evidence on the effect of urolithin A on sleep-relevant pathways, which may provide plausibility and mechanistic support for future studies designed to study sleep directly. In this context, urolithin A may affect sleep through indirect and direct mechanisms.

### 4.1 Indirect evidence

One of the most promising indirect mechanisms is through the gut microbiota (41), which has been implicated in sleep regulation via the gut–brain axis (42, 43). Experimental studies in humans have shown that sleep disturbances such as circadian rhythm misalignment (44) and sleep deprivation (45) lead to gut microbiota dysbiosis, with a decrease in microbial diversity, an increase in Firmicutes/Bacteroidetes ratio, and a decrease in short-chain fatty acid production. Similar dysbiosis was also reported in sleep disorders such as obstructive sleep apnea (46) and insomnia (47). Conversely, interventions targeting the gut microbiota, such as treatment with prebiotics, have shown positive results in sleep outcomes in animal models (48–51). Animal studies have reported that urolithin A can modulate gut microbiota composition and improve intestinal barrier function (52–55) in models of intestinal inflammation. In the context of sleep, Zhu et al. (37) reported that treatment with urolithin A can alleviate community and functional impairments in the gut microbiota and preserve intestinal barrier function in sleep-deprived mice. Interestingly, core clock genes can affect circadian rhythmicity and composition of the gut microbiota, with studies in *Bmal1*- (56) and *Per1/2*-deficient mice (57) reporting abolishment of diurnal microbial oscillations and a shift toward a more proinflammatory gut microbiota profile. Importantly, disruption of sleep–wake cycles using a jetlag model recapitulated the effect of *Per1/2* deletion on gut microbiota rhythmicity and homeostasis (57), highlighting the bidirectional relationship between sleep and gut microbiota and the mediating role of circadian mechanisms.

Urolithin A may also alleviate the inflammatory response induced by poor sleep. Sleep disruption increases neuroinflammation, especially in the hippocampus, with chronic sleep disruptions worsening inflammatory responses in the brain (40, 58, 59). Indeed, Misrani et al. (38) reported microglia and astrocytes overactivation, increase in proinflammatory cytokines, mitochondrial dysfunction, and abnormal neuronal morphology in the hippocampus of young and old mice subjected to 24 h of sleep deprivation, whereas treatment with urolithin A protected against these adverse effects. Additional studies using murine models of neurodegenerative diseases and lipopolysaccharide-induced inflammation provide further support for the protective neuroinflammatory effect of urolithin A (60–65). Interestingly, studies that included a mechanistic investigation have implicated Sirtuin 1 (SIRT1) as a mediator of urolithin A’s effect (62, 64, 65). Although there is no evidence of direct SIRT1 agonism, urolithin A is a well described mitophagy activator (63, 66, 67), contributing to improved mitochondrial efficiency and redox balance, which can indirectly improve SIRT1 expression and activity (68). Importantly, SIRT1 is directly involved in central clock regulation via modulation of transcriptional activity and stability of Per2 and PGC1α-mediated *Bmal1* expression (69), and SIRT1 signaling disruption has been associated with sleep–wake cycle changes in aging (70, 71). Thus, urolithin A may help preserve circadian rhythm robustness and sleep–wake cycle regulation via indirect SIRT1 activation under conditions of exacerbated oxidative stress and inflammation.

### 4.2 Direct evidence

Regarding direct mechanisms, urolithin A may have potential modulatory effects on central clock genes (*Clock*, *Bmal1*, *Per*, *Cry*), a pathway partially supported by existing preclinical data. Although the studies testing the effect of urolithin A on the circadian clock included in this review (33–35) did not include sleep-related outcomes, central clock gene oscillations in the SCN govern sleep–wake cycles and are, therefore, mechanistically relevant to sleep. These studies (33–35) provide preliminary evidence that urolithin A can affect central clock components in the brain, potentially strengthening SCN output to regulate sleep architecture and stability, circadian timing, and sleep–wake cycling. Further, *in vitro* evidence (72) shows that urolithin A and its phase-II metabolites can cross the blood–brain barrier, an ability that would be necessary for their direct effects in the brain. Nonetheless, while circadian clock modulation can have effects on sleep timing and structure, the effects of this modulation on sleep require direct measures, such as electroencephalogram, actigraphy, and/or polysomnography.

### 4.3 Hypothesis-only evidence

**Figure 1:**
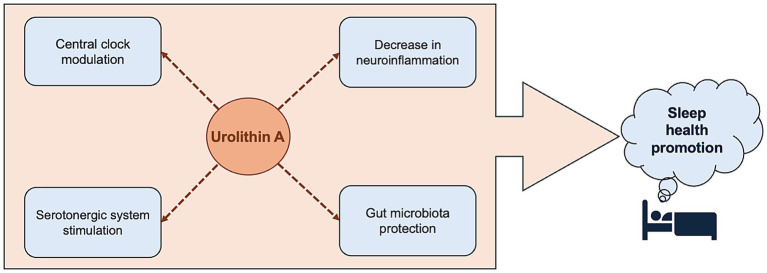
Urolithin A may modulate sleep-relevant pathways through direct and indirect mechanisms, including central clock modulation, serotonergic system stimulation, neuroinflammation attenuation, and gut microbiota protection. Dashed arrows denote potential mechanisms supported by preclinical evidence.

Urolithin A may also have a modulatory effect on metabolites involved in sleep promotion and regulation. For example, urolithin A can increase the expression of tryptophan hydroxylase-2 (TPH2) in differentiated rat serotonergic raphe cells via an increase in the transcriptional activity of 1,25-dihydroxyvitamin D_3_ (73), the active form of vitamin D. TPH2 is involved in the metabolism of tryptophan to serotonin; indeed, an increase in *TPH2* expression was accompanied by a 3- to 4-fold increase in serotonin concentration in culture medium (73). As serotonin has been shown to have critical functions in sleep (74), with disruption of the serotonergic system leading to short sleep duration and altered homeostatic sleep drive (75), urolithin A may improve sleep by stimulating the serotonergic system. As a caveat, the urolithin A doses (10–20 μM, equivalent to about 2.3–4.6 μg/mL) utilized in the study were much higher than levels achieved in human circulation (<1 μM or <0.5 μg/mL). Nonetheless, this potential mechanism merits further investigation in the context of sleep disorders such as insomnia. Figure 1 summarizes the proposed theoretical framework for the effect of urolithin A on pathways relevant to sleep health.

## 5 Future directions and conclusion

Although studies directly investigating the effects of urolithin A on sleep are lacking, there is mechanistic plausibility supporting this potential relationship, representing a novel direction in polyphenol-derived microbial metabolite research. Given the high prevalence of sleep disturbances and sleep disorders in the US population and the considerable side effects of sleep medications, non-pharmacological alternatives to improve sleep health are critically needed.

Several outstanding questions and methodological considerations remain for future studies seeking to investigate the effect of urolithin A on sleep. For cell-based studies, it is imperative that they utilize plausible doses of urolithin A (<1 μM or <0.5 μg/mL) and its main phase II metabolite, urolithin A-glucuronide to ensure that findings can translate to human physiology. Regarding preclinical studies, there are several unexplored research opportunities for experimental animal studies using, for example, (1) chronic short sleep duration or chronic sleep fragmentation models, (2) circadian misalignment/jetlag models including a urolithin A intervention arm, and (3) germ-free mice or fecal matter transplant to investigate whether the effects of urolithin A are dependent on the microbiota. The inclusion of proper controls (negative and positive controls) as well as preventive/protective versus treatment (i.e., treatment with urolithin A before versus after exposure) of sleep disturbances and disorders designs should be considered to evaluate potential benefits of urolithin A in the prevention and management of sleep conditions and their downstream adverse health effects.

Regarding clinical studies, well-designed clinical trials are needed to investigate the effect of urolithin A on sleep in humans. These trials should utilize a combination of objective and self-reported measures of sleep, such as actigraphy and sleep-related questionnaires, to capture the multidimensionality of sleep health. Including circadian rhythm markers such as dim light melatonin onset would strengthen the informative value of these trials, where feasible. Urolithin A has been shown to be safe and well tolerated in adults (21, 76), offering a unique opportunity to utilize an isolated polyphenol-derived microbial metabolite in supraphysiological doses that could not be achieved by dietary means alone. Finally, studies using isolated urolithin A or its parent polyphenols should consider individuals’ metabotypes as systemic factors associated with each metabotype may affect intervention responsiveness, such as stratifying participants by metabotype at baseline.

## Supplementary material

SUPPLEMENTARY FIGURE 1 Flow diagram of studies identified in our literature search (PubMed, Scopus, and Ovis) and included in this mini-review.
