# Supplementary figures and images for: Potential impact of urolithin A on pathways relevant to sleep health: a mini review
Source: Front Nutr. 2026 Mar 4;13:1779855. doi: 10.3389/fnut.2026.1779855 (PMC12995754; doi:10.3389/fnut.2026.1779855)

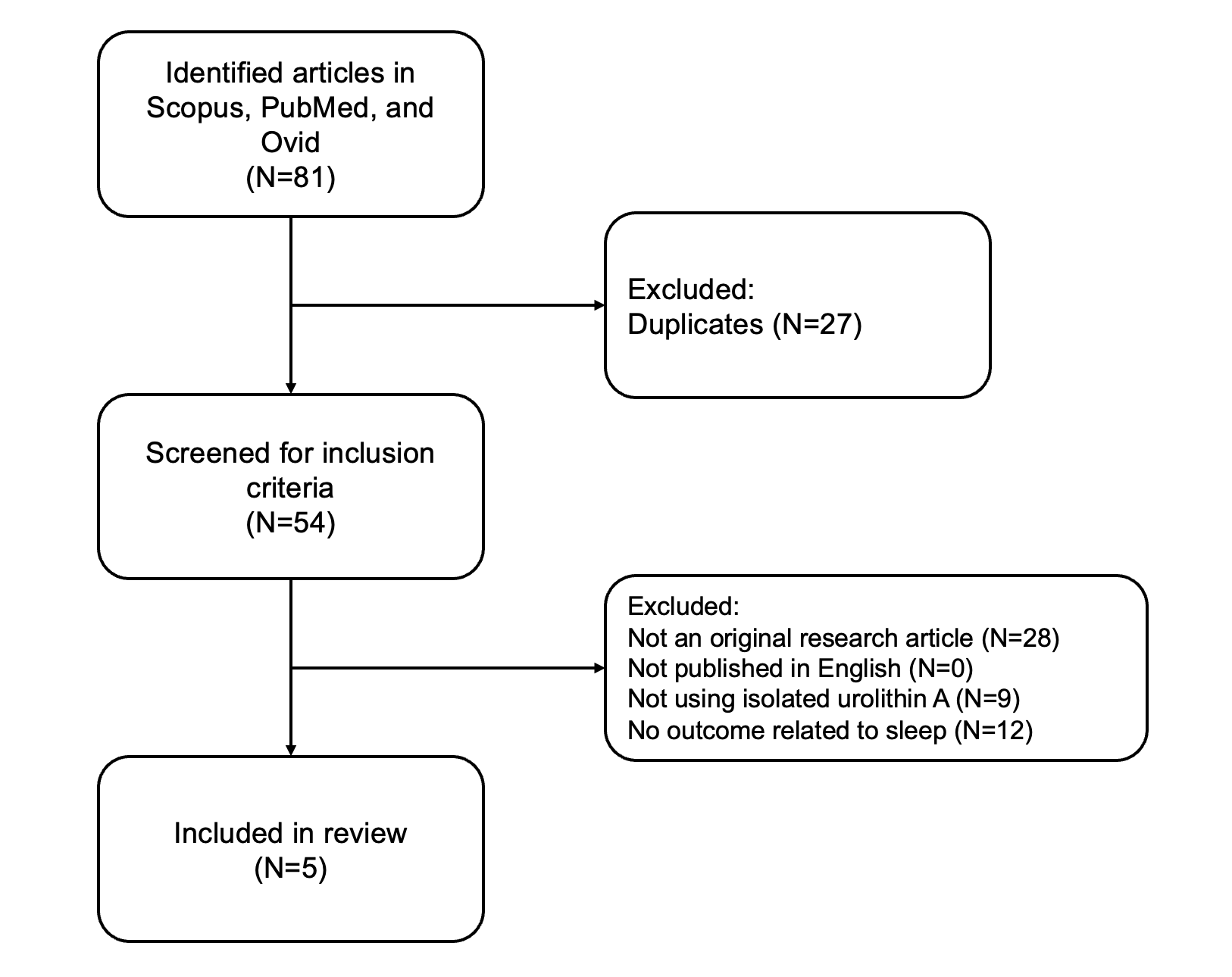

Supplement: SUPPLEMENTARY FIGURE 1 — Flow diagram of studies identified in our literature search (PubMed, Scopus, and Ovis) and included in this mini-review. [file Image_1.tiff]
